# Complete Genome Sequences of 12 Quinolone-Resistant Escherichia coli Strains Containing *qnrS1* Based on Hybrid Assemblies

**DOI:** 10.1128/MRA.01190-20

**Published:** 2021-01-28

**Authors:** Håkon Kaspersen, Thomas H. A. Haverkamp, Hanna Karin Ilag, Øivind Øines, Camilla Sekse, Jannice Schau Slettemeås

**Affiliations:** a Department of Animal Health and Food Safety, Norwegian Veterinary Institute, Oslo, Norway; b Department of Analysis and Diagnostics, Norwegian Veterinary Institute, Oslo, Norway; University of Maryland School of Medicine

## Abstract

In total, 12 quinolone-resistant Escherichia coli (QREC) strains containing *qnrS1* were submitted to long-read sequencing using a FLO-MIN106 flow cell on a MinION device. The long reads were assembled with short reads (Illumina) and analyzed using the MOB-suite pipeline. Six of these QREC genome sequences were closed after hybrid assembly.

## ANNOUNCEMENT

The presence of quinolone-resistant Escherichia coli (QREC) in the animal reservoir is a potential public health concern, especially related to plasmid-mediated quinolone resistance genes, as they might spread to more pathogenic bacteria. The *qnrS1* gene is known to be situated on plasmids with different incompatibility (Inc) groups (1, 2). Here, we aimed to select QREC strains encoding *qnrS1* on plasmids with different Inc groups to complete circular plasmid contigs.

We previously sequenced 280 QREC isolates from broilers, pigs, red foxes, and wild birds, collected through the NORM-VET program from 2006 to 2017, using short-read sequencing (Illumina, San Diego, CA) (3). The samples were either selectively isolated on MacConkey agar containing 0.06 mg/liter ciprofloxacin or randomly collected from E. coli isolated on MacConkey agar. In total, 12 QREC isolates encoding *qnrS1* from these four animal species were selected for long-read sequencing. Here, we report the hybrid assembly of these isolates, including six closed genome sequences. The hybrid assemblies were further analyzed using MOB-suite (4).

Extraction of genomic DNA was performed using the Genomic-tip 100/G kit (Qiagen, Hilden, Germany). Bacteria were enriched overnight at 37°C in 2 to 3 ml heart infusion broth (Difco, Omagh, UK). The DNA concentration was determined using the Qubit double-stranded DNA (dsDNA) broad-range (BR) assay kit (Thermo Fisher Scientific, Waltham, MA, USA), and the DNA was quality assessed using a NanoDrop One spectrophotometer (Thermo Fisher Scientific). Approximately 400 ng of high-quality DNA was subjected to library preparation using a rapid barcoding kit (SQK-RBK004; Oxford Nanopore Sequencing [ONT], Oxford, UK). Four samples were run with smaller amounts (104, 154, 324, and 369 ng), as only a maximum volume of 7.5 μl of template was allowed into the library preparation reaction. The constructed libraries were indexed using barcodes RB1 to RB12, loaded onto a FLO-MIN106 flow cell on a MinION device (Oxford Nanopore Sequencing), and run for 40 h. The raw sequence data were base called separately after the run using Guppy v.3.4.5 (5) and demultiplexed using qcat v.1.1.0 (ONT, https://github.com/nanoporetech/qcat). The sequence quality of the demultiplexed data sets was checked with NanoPlot v.1.30.0 (6). Default parameters were used for all software unless otherwise specified.

Canu v.1.9 (7) was used to improve the accuracy of the long reads, followed by Filtlong v.0.2.0 (https://github.com/rrwick/Filtlong) to remove reads of <1,000 bp from the corrected long reads. Hybrid assemblies were generated using Unicycler v.0.4.8 (8), followed by Prokka v.1.14.5 (9) to annotate the hybrid assemblies. The GC content of each assembly was calculated using the EMBOSS v.6.6.0 (10) commands “union” and “infoseq.” MOB-suite v.1.4.9 (4) was used to predict plasmid sequences from the hybrid assemblies and identify their respective replicon types. Each plasmid FASTA file generated by MOB-suite was subjected to ResFinder v.4.0 (11), VirulenceFinder v.2.0 (12), and PlasmidFinder v.2.1 (13). Plasmids containing *qnrS1* were confirmed by genome annotation with Prokka. The Illumina reads were mapped back to the assembly using BWA v.0.7.17 (14), and the depth of coverage was calculated using SAMtools v.1.10 (15) using the depth (genome-wide) and coverage (replicon) options.

The characteristics and accession numbers are presented in Table 1. The plasmid assemblies with Inc groups that allowed further typing were run on pMLST v.2.0 (13) on the Center for Epidemiology Genomics website to further determine the respective replicon types.

**TABLE 1:** Characteristics and accession numbers of the quinolone-resistant *Escherichia coli qnrS1* strains

Strain	Plasmid Inc type (pMLST)	ST^*a*^	No. of Illumina reads for:		Data for Nanopore reads:		No. of contigs	Total size (Mbp)	Replicon size (bp)	GC content (%)	No. of genes	Coverage (×)	ENA accession no. for:	
			Read 1	Read 2	No. of reads	Avg length (bp)							Raw reads	Assembly
2015-01-2097		1421	818,798	865,881	331,312	5,292.7	2^*c*^	4.68		50.8	4,507	275.1	ERR4592247	LR881940.1
	IncX1^*b*^								21,374	44.5	28	437.9		LR881941.1
2015-01-466		10	761,941	819,989	211,523	5,227.7	5^*c*^	4.87		50.6	4,695	247.4	ERR4592248	LR882052.1
	IncF (F-:A1:B1)								113,096	52.6	137	256.3		LR882053.1
	IncH								87,822	47.9	96	164.3		LR882054.1
	IncF^*b*^ (F2:A-:B-)								50,909	53.0	63	316.1		LR882055.1
	IncX1								46,065	40.7	55	294.5		LR882056.1
2016-02-324		7036	654,152	713,188	258,316	4,232.1	2^*c*^	4.90		51.0	4,656	213.1	ERR4592249	LR882050.1
	IncF^*b*^ (F-:A-:B53)								94,955	52.8	108	225.2		LR882051.1
2016-02-418		58	596,773	650,657	174,481	2,309.8	29	4.96		50.8	4,786	191.0	ERR4592250	CAJGEF01
	IncX1^*b*^								46,447^*d*^	42.9	55	310.5		
2016-02-522		1011	795,118	867,426	166,584	4,176.7	4	4.94		50.6	4,596	255.6	ERR4592251	CAJGEG01
	IncY^*b*^								78,634	50.3	103	244.5		
2016-02-620		694	676,465	740,782	438,687	3,794.8	5	4.71		50.8	4,494	227.8	ERR4592252	CAJGEH01
	IncX3^*b*^								44,425	46.3	59	251.6		
2016-17-164		7593	654,299	713,350	588,805	2,983.2	8	4.93		50.8	4,672	211.0	ERR4592253	CAJGEI01
	IncF^*b*^ (F89:A-:B53)								118,361	50.1	133	106.0		
2016-17-292		23	695,093	720,319	310,224	5,196.4	3^*c*^	4.99		50.4	4,849	217.5	ERR4592254	LR882493.1
	IncF (F24:A-:B1)								97,083	48.7	99	121.3		LR882494.1
	IncI2								59,944	42.1	83	136.4		LR882495.1
2016-17-363		48	761,196	825,502	404,780	2,644.6	5	4.67		50.7	4,478	258.2	ERR4592255	CAJGWN01
	IncH^*b*^ (unknown)								86,214	48.5	100	221.7		
2016-17-550		2165	988,537	1,058,892	218,828	4,398.6	2^*c*^	4.82		50.8	4,559	326.5	ERR4592256	LR883965
	IncY^*b*^								104,732	48.0	118	128.2		LR883966
2015-01-2838		117	388,306	418,338	129,950	3,457.6	15	5.14		50.7	4,899	98.0	ERR4592257	CAJGWP01
	IncX2^*b*^								39,630	46.0	50	337.3		
2014-01-7375		453	472,494	482,585	209,994	4,667.7	5^*c*^	5.27		50.6	5,119	34.1	ERR4592258	LR882057.1
	IncI1								98,997	49.4	110	62.5		LR882058.1
	IncF (F-:A-:B56)								82,142	47.8	89	46.4		LR882059.1
	IncX1^*b*^								47,686	43.1	56	64.0		LR882060.1
	IncF (F-:A-:B114)								42,660	52.5	54	88.3		LR882061.1

a ST, sequence type.

b Plasmid with *qnrS*.

c Genome closed.

d Plasmid not circularized.

### Data availability.

All data sets are deposited in ENA under accession number PRJEB40547 (Table 1).
